# Genomic alterations and molecular subtypes of gastric cancers in Asians

**DOI:** 10.1186/s40880-016-0106-2

**Published:** 2016-05-09

**Authors:** Xiang S. Ye, Chunping Yu, Amit Aggarwal, Christoph Reinhard

**Affiliations:** Lilly (China) R&D Center, Building 8, No 338, Jia Li Lue Road, Zhanghai Hi-Tech Park, Shanghai, 201203 P.R. China; Lilly Research Laboratories, Lilly Corporate Center, Eli Lilly and Company, Indianapolis, IN 46258 USA

**Keywords:** Gastric cancer, Cancer genome, Molecular subtyping, Heterogeneity, Oncogenic drivers, Targeted therapy

## Abstract

Gastric cancer (GC) is a highly heterogenic disease, and it is the second leading cause of cancer death in the world. Common chemotherapies are not very effective for GC, which often presents as an advanced or metastatic disease at diagnosis. Treatment options are limited, and the prognosis for advanced GCs is poor. The landscape of genomic alterations in GCs has recently been characterized by several international cancer genome programs, including studies that focused exclusively on GCs in Asians. These studies identified major recurrent driver mutations and provided new insights into the mutational heterogeneity and genetic profiles of GCs. An analysis of gene expression data by the Asian Cancer Research Group (ACRG) further uncovered four distinct molecular subtypes with well-defined clinical features and their intersections with actionable genetic alterations to which targeted therapeutic agents are either already available or under clinical development. In this article, we review the ACRG GC project. We also discuss the implications of the genetic and molecular findings from various GC genomic studies with respect to developing more precise diagnoses and treatment approaches for GCs.

## Background

Gastric cancer (GC) is the fourth most common cancer and the second leading cause of cancer death in the world [1, 2]. Although GC incidence has decreased in the developed world in the last several decades, the incidence in developing countries, particularly in Asian countries, continues to rise. Annually, nearly one million new cases are diagnosed, and 72,000 people die of GC. Gastric cancer in China alone accounts for over 40% of all new cases in the world, and the mortality in China is several times higher than the global average [3–5]. The GC incidence is anticipated to continuously increase over the next 40 years in China as the population ages.

Gastric cancers show high heterogeneity and different pathobiology across geographic regions, ethnicities, and genders, which likely reflect the different etiologies leading to GC development [6, 7]. GC is traditionally classified into two main histological subtypes, diffuse and intestinal, based on anatomic locations at the proximal and distal stomach regions, respectively. Intestinal GCs are often associated with *Helicobacter pylori* infection, unhealthy diet, and tobacco smoking, which are common in developing Asian countries [7]. On the other hand, diffuse GCs tend to be associated with genetic abnormalities [6]. Most GC cases in developed countries are diagnosed as the diffuse subtype, whereas most cases in Asian countries belong to the intestinal subtype [6]. The heterogeneity of GCs is further reflected by the lack of universally accepted treatment options in the world. Different countries typically adopt different treatment regimens, and several classification schemes for GCs are proposed [6, 7].

Over the last several decades, efforts to improve the clinical outcomes for GC patients, such as early detection through the national screening program and radical surgery, have improved the prognosis of GC in Japan and Korea, where a high GC incidence warrants a nation-wide screening program [6]. Currently, complete surgical resection represents the only potential curative treatment for early-stage GCs. However, the majority of GC patients present with advanced diseases at diagnosis in China and other developing countries that lack an advanced healthcare infrastructure, particularly in rural areas. Despite efforts in treatment standardization, chemotherapy and radiotherapy have not significantly improved the 5-year survival rate for patients with advanced (stages III and IV) diseases [1, 8]. This lack of significant progress in chemotherapy and radiotherapy is not entirely surprising as these therapies are typically indiscriminate in their activity against proliferating cells; also, these therapies were developed with no consideration of cancer heterogeneity. Individual GCs demonstrated high heterogeneity, at both the histological and molecular levels, in genomic studies. This heterogeneity undoubtedly plays an important role not only in disease progression but also in the response to therapy and subsequent emergence of resistance. For instance, in a subpopulation of GC patients with Erb-B2 receptor tyrosine kinase 2 (*ERBB2*) amplification, the addition of trastuzumab to standard 5-fluorouracil and platinum chemotherapy significantly improved the overall survival [9]. This finding underscores the importance of, as well as the need for, molecular characterization and subtyping of GCs to develop safer and more efficacious treatment options.

Recent next-generation sequencing and gene expression profiling studies on GC have begun to establish a comprehensive landscape of genomic alterations. They have also identified a range of actionable genetic drivers as drug targets or diagnostic biomarkers. Furthermore, the combination of global gene expression profiling with longitudinal clinical data has defined clinically relevant molecular subtypes of GC. In this article, we discuss the Asian Cancer Research Group (ACRG) GC project and highlight significant findings and molecular insights that can be used for developing more effective targeted therapies and accurate diagnostic approaches for GC.

### Genomic alterations in GCs in Asians

To dissect the genomic basis and underlying genetic heterogeneity of GCs, the ACRG selected a diffuse GC-enriched cohort from Samsung Medical Center, Korea. The ACRG then performed whole-genome sequencing (WGS) on 49 cases of advanced stage tumors (stage IV, 19 cases; stage III, 29 cases; and stage II, 1 case) with high tumor cell contents and matched peripheral blood samples [10]. Thirty-one tumor samples were diffuse, and 18 were intestinal, microsatellite stable (MSS) GC.

Deep WGS identified genetic alterations and further revealed mutation heterogeneity and differences between the diffuse and intestinal subtypes of GC, providing a molecular basis for their differing pathobiology and prognosis. The number of somatic variants in the individual cancer genome varies greatly, ranging from 172 to 38,328 with a median of 9036 variants per tumor. A total of 4528 somatic mutations in 2553 genes were detected, with 384 genes mutated in at least two tumors. An analysis of significantly mutated genes confirmed known mutations in GCs that had been previously identified, such as tumor protein 53 (*TP53*), AT-rich interactive domain 1A (*ARID1A*), transforming growth factor beta receptor 2 (*TGFβR2*), and cadherin 1 (*CDH1*). The analysis also uncovered new significantly mutated genes, including spectrin repeat-containing, nuclear envelope 1 (*SYNE1*; *n* = 10, 20%) and transmembrane protease, serine 2 (*TMPRSS2*; *n* = 3, 6%) in this Korean cohort [10]. The biological significance of the *SYNE1* and *TMPRSS2* mutations in GCs remains to be explored experimentally and clinically. Recurrent *SYNE1* mutations were first identified in glioblastoma multiforme (GBM) tumors [11]. *SYNE1* polymorphism is associated with invasive epithelial ovarian cancer risk [12]. Interestingly, subsequent association analysis of somatic mutations and gene expression changes in GBM identified *SYNE1* as a major node; *SYNE1* mutations have drastic effects on the expression of 543 genes, including mismatch repair genes MutS homolog 6 (*MSH6*) and MutL homolog 1 (*MLH1*). Their effects are only second to the effects of isocitrate dehydrogenase 1 (*IDH1*) mutations [13]. Clonal analysis of the somatic variations revealed that the intestinal subtype of GC has significantly higher ploidy and clonality than the diffuse subtype [10]. The low clonality observed in the diffuse subtype of GC indicates the presence of considerable intra-tumor heterogeneity, which has clinical implications for the emergence of drug resistance. The results also provide a plausible explanation for the poor prognosis of the diffuse subtype of GC. The intra-tumor heterogeneity and clonality in GCs were also shown in a genomic profiling study of GC patients in North China that included WGS data of two GC patients, each with three primary tumors and two matching metastatic lymph nodes [14].

The intestinal subtype of GC also shows more structural variations than the diffuse subtype [10]. These changes include gene fusion, translocation, and copy number variations (CNVs). Acyl-CoA-binding domain-containing 5-zinc finger E-box-binding homeobox 1 (*ACBD5*-*ZEB1*), WD repeat-containing protein 52 (*WDR52*)-*TGFβR2*, Sine oculis-binding protein homolog-mesenchymal-epithelial transition factor (*SOBP*-*MET*), and lactation-elevated protein 1 (*LACE1*)-*MET* fusions were confirmed by reverse transcription-polymerase chain reaction (RT-PCR) and Sanger sequencing. The overexpression of epidermal growth factor receptor (*EGFR*), *MET* and *ERBB2* amplifications were validated by both fluorescent in situ hybridization and immunohistochemistry. These genetic changes were largely observed in the *TP53*-mutant background, and they are mutually exclusive. In general, cancer cells of the diffuse subtype have fewer genetic alterations and are mostly diploid. Mutations in *CDH1*, catenin alpha-1 (*CTNNA1*), or phosphatidylinositol-4,5-bisphosphate 3-kinase, catalytic subunit alpha (*PIK3CA*) are also known to be associated with the diffuse subtype of hereditary GC [10]. The high mutation load and resulting neo-antigens are implicated in the tumor immune response [15]. In agreement, recurrent programmed death-ligand (*PD*-*L*)*1* and *PD*-*L2* gene amplifications were identified, particularly in Epstein-Barr virus-positive (EBV^+^) intestinal GCs [16]. This possibility is very interesting from the medical perspective, and it has significant implications for developing effective immunotherapy for GCs. However, a recent retrospective analysis of over 1000 GC gene expression profiling data showed that GCs in Western and Asian countries differ in their immune response signatures. GCs in Western countries, which are more often of the diffuse subtype, are associated with an enrichment of tumor-infiltrating T cells [17]. Although the results of this large retrospective analysis are intriguing, they must be validated through prospective studies that are specifically designed to compare geographic effects. The ongoing development of immune checkpoint inhibitors, programmed death 1 (PD-1) and PD-L1 monoclonal antibodies, in GCs will help define how genomic alterations influence the tumor immune response.

The pathway-based analysis shows that recurrent mutations occur in various cell adhesion, axon guidance, and transforming growth factor β (TGFβ) pathways [10]. TGFβ signaling regulates cell proliferation and suppresses the immune response, and mutations in this pathway are also identified in other GC genomic studies [14, 18]. It is interesting that *TGFβR2* mutations identified by the ACRG are not inactivating mutations [10]. The significance of recurrent *TGFβR2* genetic alterations in the context of immunotherapy needs to be evaluated further. Axon guidance molecules are implicated in regulating cell migration and apoptosis in cancer [19]. Mutations of these molecules were previously observed in pancreatic ductal adenocarcinoma [20]. In this Korean GC cohort, axon guidance pathway mutations in ephrins, netrins, semaphorins, and slits were highly prevalent and observed in 59% (*n* = 29) of the analyzed tumors [10]. Mutually exclusive mutations in the ephrins and slit glycoprotein/roundabout receptor (SLIT/ROBO) pathway genes indicate that they are driver mutations in cancer development. Inactivation of slit homolog 2 (*SLIT2*) by RNA interference (RNAi) promotes GC cell growth that is mediated through activation of protein kinase B/catenin, beta 1 (AKT/CTNNB1) signaling [21]. The axon guidance signaling pathway is a potential novel cancer therapeutic target.

### Other genomic studies of GC

As summarized in Table 1, a growing list of genomic studies, including the ACRG project, have been carried out independently [10, 14, 16–18, 22–31]. The majority of these studies have focused on GCs in Korea [10, 17, 22–25], China [14, 17, 18, 26], Singapore [17, 22, 27], and Japan [28], reflecting the medical significance of GCs in these countries. The molecular findings from these studies confirm the genetic heterogeneity of GCs. They also identify a common set of genetic alterations, such as *CDH1* and Ras homolog family member A (*RhoA*) mutations, that are associated with the diffuse subtype of GCs from different geographic regions, indicating that the cancers share a common genetic origin. As *CDH1* and *RhoA* regulate cell motility, mutations in these genes offer a mechanistic basis for the highly malignant phenotype of poorly differentiated cells, including prominent infiltration and stromal induction in diffuse GCs. These genomic findings are consistent with observations that the diffuse subtype of GC is associated with genetic abnormalities, and some of these are hereditary. On the other hand, the intestinal subtype of GC is more associated with environmental factors.

**Table 1:** Summary of genomic studies on gastric cancer (GC)

Reference	Sample size	Ethnicity	Histology (Lauren’s type)				Technology platform							Molecular subtype
			Intestinal	Diffuse	Mixed	Not specified	WES	WGS	RNAseq	SNP/copy number microarray	Targeted sequencing	Gene expression microarray	DNA methylation profiling	
[10]	49 (MSS only)	Korean	18	31	0	0		√						NA
[14]	294	Northern Chinese	139	155	0	0	√	√			√			High clonality and low clonality
[16]	75	Asian	196	69	19	11	√	√	√	√			√	EBV^+^, MSI, GS, and CIN
	220	Non-Asian												
[17]	890	Asian	0	0	0	1016						√		NA
	126	Non-Asian												
[18]	100	Chinese (Hong Kong)	57	29	14	0		√		√		√	√	NA
[22]	386	Singaporean	253	183	82	3						√		Genomic intestinal and genomic diffuse
	65	Korean												
	70	Australian												
[23]	19	Korean	5	14	0	0	√	√						NA
[24]	300	Korean	150	142	8	0				√	√	√		MSS/TP53^−^, MSS/TP53^+^, MSS/EMT, and MSI
[25]	103	Korean	61	36	5	1	√			√				NA
[26]	22	Chinese (Hong Kong)	18	2	2	0	√							NA
[27]	15	Singaporean	11	3	1	0	√			√		√		NA
[28]	87	Japanese	0	87	0	0	√				√			NA
[29]	36	Non-Asian	12	10	14	0						√		Proximal non-diffuse, diffuse, and distal non-diffuse
[30]	17	Non-Asian	0	0	0	51	√		√	√				NA
	34	Vietnamese												
[31]	116	Non-Asian	12	24	0	80					√			NA

*WES* whole-exome sequencing, *WGS* whole-genome sequencing, *RNAseq* RNA sequencing, *SNP* single-nucleotide polymorphism, *MSS* microsatellite stable, *NA* not available, *EBV*, Epstein-Barr virus, *MSI* microsatellite instability, *GS* genomically stable, *CIN* chromosomal instability, *TP53* tumor protein 53, *EMT* epithelial-to-mesenchymal transition

However, genomic studies on GCs from China, Korea, Japan, and Russia also identified non-overlapping, significantly mutated genes or altered pathways in different cohorts. For example, the ACRG study identified *SYNE1* mutations in 20% of GCs as a significantly mutated cancer gene [10], which has interesting biological implications as discussed above. A study of another Korean cohort discovered B cell lymphoma 2 like 1 (*BCL2L1*) amplification in 18.4% and deletion in liver cancer 1 (*DLC1*) mutations in 10.9% of GC cases [25]. The genomic alterations in *BCL2L1* and *DLC1* influence drug sensitivity in GCs. *BCL2L1* amplification confers sensitivity to the BLC2L1 inhibitor when used in combination with chemotherapeutic agents. *DLC1* mutations promote activation of Rho/Rho-associated protein kinase (ROCK) kinase activity and make cells sensitive to ROCK kinase inhibitors [25]. In addition, a study performed on a cohort from North China identified frequent mutations in the neuregulin 1 (*NRG1*) and *ERBB4* genes [14]. Novel genomic alterations identified by different studies further demonstrate the complexity and heterogeneity of GCs, which also prompts the speculation that additional genomic alterations remain to be identified. It should be noted that the sample sizes in these GC genomic studies are small; the samples typically include fewer than 100 cases. Only the Cancer Genome Atlas (TCGA) project profiled a larger cohort of patients (*n* = 295); 75% of the patients were Caucasians (Russians, Americans, Poles, Ukrainians, and Germans), and the remaining patients were Asians (South Koreans and Vietnamese) [16]. In addition to difficulty with small sample sizes, differences in the genomic technology platforms and bioinformatics pipelines may have contributed to the differences in the genomic alterations identified among the studies. Indeed, Li et al. [32] performed a retrospective, integrated analysis of 544 GC genomic data samples from previous genomic studies using an improved bioinformatics pipeline to identify significantly mutated genes. This analysis identified six previously unreported, significantly mutated genes and 12 recurrent mutated genes that exhibit higher prevalence than previously realized. These findings suggest that more profiling studies that evaluate large sample sizes for each patient population using the same advanced genomic profiling platform and bioinformatics pipeline may be needed to comprehensively determine and characterize genomic alterations in different GC cohorts.

### Molecular subtyping of GCs in Asians

The high molecular heterogeneity of GCs, as demonstrated in genomic studies, further underscores the need for molecular subtyping of GCs to improve the prognosis, diagnosis, and treatment outcomes. Molecular subtyping of heterogeneous tumors, such as breast and lung cancers, has shown tremendous clinical benefits and has redefined treatment practices. Breast cancer was initially classified into four major subtypes, luminal, human epidermal growth factor 2 (HER2)-enriched, basal-like, and normal breast-like, based on the gene expression patterns according to cDNA microarrays [33]. The subtypes were subsequently further refined, and they are currently known as luminal A, luminal B, HER2-enriched, and basal-like subtypes [34]. In recent years, five novel gene expression prognostic tests for breast cancer were developed. These tests provide more reliable and reproducible results than immunohistochemistry-based assays with respect to treatment option selection [34]. The significantly mutated genes and pathways in these subtypes have further guided the development of therapies that are targeted against these genetic alterations.

In addition to the aforementioned WGS study of 49 gastric tumor specimens that assessed the mutational landscape of GC, the ACRG characterized 251 additional primary gastric tumors by gene expression profiling, genome-wide copy number microarrays, and targeted gene sequencing to identify and define clinically relevant molecular subtypes through integrated analysis of genomic alterations, survival outcome, and recurrence data [24]. The ACRG obtained 300 primary GC tumor specimens from Samsung Medical Center, which were selected on the basis of over 60% histological purity and availability of long-term follow-up data. Principle component (PC1-3) analysis was performed on the expression data, and the results were compared with a small pre-defined set of gene expression signatures for the epithelial-to-mesenchymal transition (EMT), microsatellite instability (MSI), cytokine signaling, cell proliferation, DNA methylation, TP53 activity, and the normal gastric tissue [35]. The analysis classified the 300 gastric tumor specimens into the following four molecular subtypes: MSI (*n* = 68), MSS/EMT (*n* = 46), MSS/TP53^+^ (*n* = 79), and MSS/TP53^−^ (*n* = 107). The TCGA also classified GCs into four subtypes, including EBV^+^, MSI, genomically stable (GS), and chromosomal instability (CIN). Importantly, the ACRG subtype classification was also reproduced in the gene expression datasets of the TCGA cohort [16] and a Singapore cohort [36], although the proportion of each molecular subtype varied across the datasets. This variation may reflect the known geographic heterogeneity of GCs.

The ACRG molecular subtypes are associated with distinct clinical features of GC [24]. The majority of MSS/EMT subtype GC cases (>80%) were diagnosed as the diffuse type at stage III/IV and occurred at significantly younger ages than the other subtypes. On the other hand, the MSI subtype occurred predominantly at the antrum (75%), and over 60% were diagnosed as the intestinal subtype and at an early stage (I/II). In addition, EBV infection occurred more frequently in the MSS/TP53^−^ group than in other groups. Survival analysis showed a substantial difference among four molecular subtypes; the MSS/EMT subtype showed the worst prognosis, and the MSS subtype showed the best prognosis, which was followed by the MSS/TP53^+^ and MSS/TP53^−^ subtypes. Furthermore, the MSS/EMT group also showed a higher rate of recurrence than the MSI group. The MSS/EMT group had a higher percentage (64%) of the first site of recurrence with peritoneal seeding and a lower percentage of liver metastasis (4.6%) than other groups. A comparison of the ACRG subtypes [24] with the TCGA subtypes [16] showed similarities in the tumors with MSI and an enrichment of the TCGA GS, EBV^+^, and CIN subtypes in the ACRG MSS/EMT, MSS/TP53^+^, and MSS/TP53^−^ subtypes when applied to both datasets. However, the TCGA CIN and GS subtypes were present in all ACRG subtypes in the ACRG dataset. The TCGA cohort had a lower percentage of diffuse subtype cases than the ACRG cohort (24% in TCGA vs. 45% in ACRG). Interestingly, the majority of the TCGA diffuse subtype cases (57%) were present in the TCGA GS subtype, but only 27% of the cases were present in the ACRG MSS/EMT subtype, suggesting that the TCGA diffuse subtype cases were less heterogeneous. The analysis suggests that the ACRG molecular classification of GCs is unique and clinically relevant.

Targeted sequencing and CNV analysis revealed that molecular subtypes are associated with prevailing somatic alterations, many of which are clinically relevant and actionable [24]. The MSI subtype exhibited hyper-mutation with prevalent mutations in the Kirsten rat sarcoma viral oncogene homolog (*KRAS*; 23.3%), phosphoinositide 3 kinase-phosphatase and tensin homolog-mechanistic target of rapamycin (PI3K-PTEN-mTOR) pathway genes (50.6%), anaplastic lymphoma kinase (*ALK*; 16.3%), and *ARID1A* (44.2%). By contrast, the MSS/EMT subtype exhibited a lower number of mutations. The MSS/TP53^−^ subtype had more CNVs and was associated with recurrent focal amplification in *ERBB2*, *EGFR*, Cyclin E1 (*CCNE1*), Cyclin D1 (*CCND1*), murine double minute 2 (*MDM2*), roundabout homolog 2 (*ROBO2*), GATA-binding protein 6 (*GATA6*), and v-Myc avian myelocytomatosis viral oncogene homolog (*MYC*). Importantly, *ERBB2*, *EGFR*, *CCNE1*, and *CCND1* amplifications are mutually exclusive, indicating that they are driver alterations. The identification of genomic alterations and molecular subtypes, as performed in the genomic studies discussed in this article, provides important biological insight into the biology of GCs. The insight then helps guide the development of targeted therapies that can effectively treat subtypes of GC (Table 2).

**Table 2:** Summary of clinically relevant and actionable genomic alterations and molecular subtypes of GC

Subgroup	Key mutations	Key CNV events	Drugs in development
MSI	KRAS, PIK3CA (H1047R), PTEN, mTOR, ARID1A	Very few alterations	MEK-ERK and PI3K-mTOR pathway inhibitors, immunotherapies
MSS/EMT	PIK3CA, RhoA	CCNE1	PI3K-mTOR, CDK2, and ROCK inhibitors
MSS/TP53^−^	TP53	CCND1, CCNE1, ERBB2, EGFR, KRAS, MYC	RTK-focused agents, MEK-ERK, CDK4/6, and CDK2 inhibitors
MSS/TP53^+^	ARID1A, PIK3CA (E542/545K)	CCNE1, KRAS	MEK-ERK, CDK2, and PI3K-mTOR pathway inhibitors

*CNV* copy number variation, *KRAS* kirsten rat sarcoma viral oncogene homolog, *PIK3CA* phosphatidylinositol-4,5-bisphosphate 3-kinase, catalytic subunit alpha, *PTEN* phosphatase and tensin homolog, *mTOR* mechanistic target of rapamycin, *ARID1A* AT-rich interactive domain 1A, *MEK* mitogen-activated protein kinase kinase, *ERK* extracellular signal-regulated kinase, *PI3K* phosphoinositide 3 kinase, *RhoA* Ras homolog family member A, *CCNE1* cyclin E1, *CDK* cyclin-dependent kinase, *ROCK* Rho-associated protein kinase, *CCND1* cyclin D1, *ERBB2* Erb-B2 receptor tyrosine kinase 2, *EGFR* epidermal growth factor receptor, *MYC* v-Myc avian myelocytomatosis viral oncogene homolog, *RTK* receptor tyrosine kinase. Other abbreviations as in Table 1

## Conclusions

The ACRG GC genomics project comprehensively characterized 300 patient tumors with longitudinal follow-up clinical data by a combination of WGS, whole-exome sequencing, CNV analysis, targeted sequencing, and gene expression profiling approaches [10, 24]. Together, the study further demonstrated the heterogeneity of GCs at the molecular and genetic levels, established a comprehensive mutational landscape, and defined clinically relevant molecular subtypes of GC with actionable oncogenic drivers. In fact, we found that approximately 80% of GCs in this Korean cohort harbor at least one clinically relevant genomic change [10]. Most frequent, clinically relevant alterations affect cell cycle/growth (*p53*, cyclin-dependent kinase inhibitor 2A [*CDKN2A*], *CCND1*, *CCNE1*, aurora kinase A [*AURKA*], cyclin-dependent kinase 6 [*CDK6*], *c*-*Myc*, and *TGFβR2*); receptor tyrosine kinase (RTK) signaling (*KRAS*, neuroblastoma RAS viral oncogene homolog [*NRAS*], *MET*, fibroblast growth factor receptor [*FGFR*], *EGFR*, *ERBB2*, *PTEN*, *PIK3CA*, and *BRaf*); DNA repair (breast cancer [*BRCA*]*1/2*, ataxia telangiectasia mutated [*ATM*], and *MDM2*); and epigenetics (*ARID1A*, mixed-lineage leukemia protein 2 [*MLL2*], and DNA-methyltransferase 2A [*DNMT2A*]). The identification of recurrent oncogenic drivers in cancer has enabled the development of a new generation of targeted cancer therapies, such as EGFR tyrosine kinase inhibitors, which have dramatically changed the treatment practices for a number of cancers [34, 37]. Many targeted cancer therapies that target these pathways are either available for other indications or under advanced clinical development. The genomic findings from these studies will inform and accelerate the development of effective targeted therapies for GCs. These genomic studies are also expected to promote comprehensive preclinical studies on molecular mechanisms underlying the pathobiology of GC in relevant disease models, such as patient-derived xenograft (PDX) models [38]. These preclinical and clinical studies will further advance our understanding of GC and accelerate the development of more precise diagnostic approaches and safer, more effective treatment options. Ultimately, personalized medicine will be used to improve the outcomes of individual GC patients. We are currently in a new, exciting era of GC research, including drug development, because of the rapid advancement in genomic technologies.
